# The Vascular Circadian Clock in Chronic Kidney Disease

**DOI:** 10.3390/cells10071769

**Published:** 2021-07-13

**Authors:** Søren Egstrand, Maria L. Mace, Klaus Olgaard, Ewa Lewin

**Affiliations:** 1 Nephrological Department B, Herlev Hospital, University of Copenhagen, 2730 Copenhagen, Denmark; soeren.egstrand.01@regionh.dk; 2 Nephrological Department P, Rigshospitalet, University of Copenhagen, 2100 Copenhagen, Denmark; maria.lerche.mace@regionh.dk (M.L.M.); olgaard@rh.dk (K.O.)

**Keywords:** vascular calcification, ccl2, inflammation, adhesion molecules, VCAM-1, ICAM-1, uremia, thrombomodulin, cardiovascular disease

## Abstract

Chronic kidney disease is associated with extremely high cardiovascular mortality. The circadian rhythms (CR) have an impact on vascular function. The disruption of CR causes serious health problems and contributes to the development of cardiovascular diseases. Uremia may affect the master pacemaker of CR in the hypothalamus. A molecular circadian clock is also expressed in peripheral tissues, including the vasculature, where it regulates the different aspects of both vascular physiology and pathophysiology. Here, we address the impact of CKD on the intrinsic circadian clock in the vasculature. The expression of the core circadian clock genes in the aorta is disrupted in CKD. We propose a novel concept of the disruption of the circadian clock system in the vasculature of importance for the pathology of the uremic vasculopathy.

## 1. Introduction

The circadian clock system plays a pivotal role in regulating all aspects of physiology; locomotor activity, cognition, behavior, metabolism, organ function, hormone secretion, immunity and the cell cycle [1,2]. It operates with a periodicity of 24 h. This is to anticipate predictable changes in the environment following the Earth’s rotation. The master pacemaker of mammalian circadian rhythmicity is in the hypothalamic suprachiasmatic nucleus (SCN) [3]. The SCN responds to light cues from the retinal ganglion cells and accordingly coordinates the circadian rhythms of the central and peripheral tissues via the neuronal, hormonal and metabolic signaling pathways. In addition to the central pacemaker in the SCN, a circadian molecular clock has further been found in peripheral tissues [4]. While the light–dark cycle is the main cue for the master clock in the SCN, feeding is the predominant cue for many of the peripheral tissue clocks [5,6,7,8].

The circadian clock is involved in the regulation of the many different aspects of vascular physiology, including blood pressure and endothelial function [9,10], and is further involved in the regulation of processes of pivotal importance for vascular health, such as glucose and lipid metabolism, macrophage polarization, inflammatory responses, redox signaling, fibrinolytic activity, platelet activation and coagulation [11,12,13,14,15,16,17,18]. In addition to the circadian rhythmicity in the parameters of physiological importance for the cardiovascular system [19,20], a number of pathological cardiovascular and thromboembolic events, including stroke, myocardial infarction and sudden cardiac arrest, exhibit a clear diurnal variation, where the onset frequently peaks during the early morning hours [21,22,23]. 

Disruption of the circadian rhythm in day/night or in the timing of food intake has been identified as a critical factor leading to cardiovascular diseases and atherosclerosis [20,24,25,26,27,28]. Epidemiological studies in humans have shown that a disturbed circadian rhythm is associated with an increased risk of metabolic and cardiovascular complications [29,30]. Night shift work leads to misalignment of the sleep–wake and fasting–feeding cycles of the endogenous circadian clock and is associated with adverse metabolic effects [31,32,33]. A difference in normal blood pressure levels between day and night is essential for cardiovascular health. Nightshift workers develop a non-dipping systolic blood pressure pattern and have an increased risk for developing hypertension and cardiovascular complications [34,35].

Sleep disorders, prevalent in patients with chronic kidney disease (CKD), are associated with the disturbed rhythm of melatonin and a decreased amplitude of this hormone, which primarily is released from the pineal gland and is involved in sleep–wake timing, blood pressure regulation and in synchronizing circadian rhythms [36,37]. The prevalence of increased blood pressure during sleep and non-dipper patterns is high in CKD patients [38,39].

The impact of kidney insufficiency on the CNS might also involve disruption of the molecular clock system [40]. Recently, it was experimentally shown that mice with CKD developed unstable behavioral circadian rhythms, fragmented sleep and disturbed locomotor activity [41], which were associated with a disturbed amplitude in the circadian rhythm of the core circadian molecule *Period 2* in the SCN, indicating that uremia affects the central circadian pacemaker in the hypothalamus [41]. 

Chronic kidney diseases are associated with extremely high cardiovascular mortality, which is related to the burden of vascular calcification [42,43]. Alterations in endothelial cells, vascular smooth muscle cells (VSMC) and periarterial cells in CKD are linked to disturbed mineral homeostasis, accelerated senescence, uremic toxins, oxidative stress, abnormal lipid metabolism and chronic inflammation [42,44,45,46]. Understanding the importance of the disrupted circadian rhythmicity for the pathophysiology of uremic vasculopathy is emerging [47,48].

Besides leading to disruption of the central molecular clock system, kidney insufficiency may also have an important impact on the different molecular circadian clocks in individual peripheral tissues and might therefore contribute to the significance of uremic symptoms [49]. Our group has recently shown that the parathyroid gland has an intrinsic molecular circadian clock, which is disrupted in CKD [48]. In the kidneys, several circadian regulations are present, resulting in circadian patterns in the renal blood flow, glomerular filtration rate, tubular transport and metabolism [50]. Kidney-specific alterations in circadian regulation are associated with changes in the handling of sodium and water and, at the same time, changes in the regulation of aldosterone levels and blood pressure, including nocturnal dippings [51,52,53,54].

In the present review, the impact of CKD on the circadian clock in the vasculature is addressed. 

## 2. The Molecular Circadian Clock

Both central and peripheral circadian clocks involve the same set of genes and are regulated by an interplay of positive and negative transcription–translation feedback loops that change dynamically during the day–night cycle [55]. 

**Figure 1:**
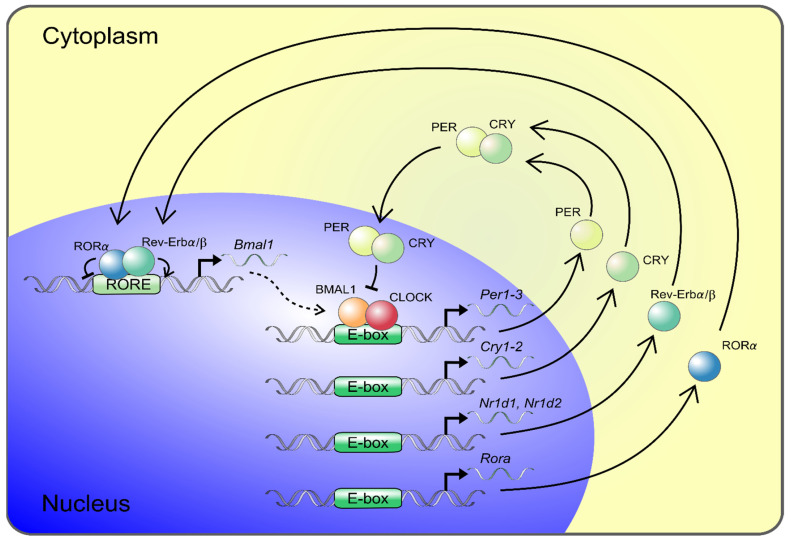
The core components of the molecular circadian clock. Transcription–translation feedback loops of the circadian clock are shown. The transcription factors BMAL1 and CLOCK are core components of the molecular circadian clock positive limb. BMAL1 and CLOCK heterodimerize and bind to E-box elements in the promoters of period 1–3 (*Per1–3*) and cryptochrome 1–2 (*Cry1–2*) genes, which constitute the negative limb in the feedback loop. PER and CRY proteins dimerize and translocate back into the nucleus, hindering CLOCK and BMAL1 transcriptional activity and thereby repress their own expression. The main loop is modulated by an accessory feedback loop driven by the BMAL1/CLOCK induction of Rev-Erbα/β and RORα mediating opposing actions and repressing and activating BMAL1 gene expression, respectively. The circadian clock components regulate the expression of clock-controlled tissue-specific output genes, and about 4% of the aorta transcriptome shows circadian rhythmicity.

Circadian locomotor output cycles kaput (CLOCK) and Brain and muscle Arnt-like protein 1 (BMAL1) represent major components of the clock’s positive limb and are the central transcription factors of the molecular circadian clock system (Figure 1). The complex of BMAL1 and CLOCK binds to E-box DNA sequences and induces the expression of several genes and proteins, including those of *Period* (*Per*) and *Cryptochrome* (*Cry*), which constitute the major arm of the negative limb (Figure 1) [56]. PER accumulates in the cytoplasm, where it forms a complex with CRY, as well as other modulator proteins, and acts as a repressor of CLOCK/BMAL1. Subsequently it inhibits its own expression, resulting in oscillation of the gene expression in a circadian manner [56]. This main loop interplays with other feedback loops, including those of REV-ERB or the retinoid acid-related orphan receptor (ROR), mediating opposing actions that are repressing or activating *Bmal1* gene expression [57,58]. The preferential feedback loop structures vary across tissues and peripheral organs, where they contribute to tissue-specific circadian rhythms. BMAL1/CLOCK controls the expression of approximately 10% of the transcripts of the genome in a tissue-specific manner [59,60]. In the aorta of mice, 4% of the protein-coding genes revealed circadian oscillation [61].

## 3. The Internal Vascular Circadian Clock and Its Role in Vascular Pathology

Potentially, disturbed molecular circadian clocks in individual peripheral tissues may, in CKD, contribute to the uremic symptoms [41,48,49,62]. Blood vessels are composed of three layers. The inner layer, intima, is mainly composed of endothelial cells responding to circulating factors, determining the vascular tissue permeability; regulating the vascular tone and regulating the factors related to coagulation, fibrinolysis and platelet adhesion. The lamina media consists mainly of smooth muscle cells taking part in vascular constriction and dilatation. The outer layer, adventitia, consists of connective tissue and contains fibroblasts. Endothelial inflammation and atypical cell differentiation are hallmarks of an atherosclerotic lesion, while cells with multilineage potential in the arterial wall—pericytes, smooth muscle cells and adventitial myofibroblasts—may all contribute to the development of vascular calcifying diseases [47]. Arterial calcification can be classified into tunica intima calcification, related to atherosclerosis, and into tunica media calcification. Media calcification is predominantly observed in systemic metabolic disorders such as CKD and diabetes mellitus and as part of the aging process. 

In the vasculature, a functional circadian clock has been demonstrated in the different types of cells in the vascular wall, and different components of the molecular circadian clock have been demonstrated in endothelial cells, in the VSMC of lamina media and in cultured fibroblasts from the adventitia of the arteries [20,63,64,65,66]. With the generation of genetically altered mice models, some important features of the intrinsic vascular circadian clock in cardiovascular pathology have been uncovered [20,67]. As such, in several animal studies, circadian rhythm disruption was achieved either by genetically modifying the molecular circadian clock in the individual cells in the cardiovascular system or by the global deletion of core molecular circadian clock genes. Global knockout mice strains have been generated for all circadian clock genes as a single knockout or in combination. Bmal1^−/−^ showed metabolic disturbances, severe accelerated aging and the dramatically reduced lifespan of an average of 8 months together with a vascular phenotype of endothelial dysfunction and an increase in neointimal area, pathological remodeling and atherosclerosis [26,68,69,70]. 

CLOCK^−/−^ mice have preserved rhythmicity in the locomotor activity but developed an aging phenotype with cataracts, dermatitis and a 15% reduced lifespan [71,72]. Per2^−/−^ mice have aortic endothelial dysfunction, involving a decreased production of NO and vasodilatory prostaglandins and increased release of cyclooxygenase-1-derived vasoconstrictors [73]. Rev-erbα^−/−^ and Rev-erbβ^−/−^ double-knockout mice showed a markedly altered circadian wheel-running behavior and deregulated lipid metabolism [74]. The CK1e mutation in the golden hamster (the Tau mutant) reduced the lifespan, which can be prolonged by almost 20% following the transplantation of wildtype SCN [75]. Both Bmal1^−/−^ and Clock-mutant mice exhibited impaired vasorelaxation in response to acetylcholine [76]. Excellent review articles have been published recently that have highlighted the importance of the circadian clock in the cardiovascular system [20,67]. 

An interesting study demonstrated the development of atherosclerosis in mice with a disrupted circadian clock (*Bmal1* or *Per2,3* double-KO) [24,77]. The transplantation of blood vessels from these animals into the wildtype littermates did not prevent this pathology and still resulted in atherosclerosis [24]. This indicates that the intrinsic vascular tissue clock has an autonomous influence in atherosclerotic disease. Deletion of vascular endothelial-specific *Bmal1* accelerated both microvascular and macrovascular injuries in mice, resulting in increased levels of oxidative injury and in the development of neointimal hyperplasia [78]. A diurnal variation in the time for a thrombotic vascular occlusion to occur subsequent to a photochemical injury in the control animals was disrupted in mice where *Bmal1* selectively was deleted in the endothelium [79]. The selective deletion of *Bmal1* in smooth muscle cells, but not in cardiomyocytes, abolished the diurnal variations in smooth muscle contractility and compromised the circadian rhythm in blood pressure without affecting the SCN-controlled locomotor activity of the mice [80]. The young animals in this model had lower basal blood pressure. Moreover, the VSMC deletion of *Bmal1* provided an effective protection from developing aortic aneurisms in two different abdominal aorta aneurism murine models [81]. 

Thus, it was clearly demonstrated that the vascular wall has an internal circadian clock with a significant impact on vascular function and pathology. 

## 4. The Vascular Circadian Clock Is Disrupted in CKD

Structural and functional changes in the vasculature are observed early in CKD, including the altered expression of genes related to the developmental program and the contractility of VSMC, vascular stiffness and endothelial dysfunction [47,82]. Dedifferentiated VSMC are susceptible to osteoblastic transformation and in more advanced stages of CKD to the progressive accumulation of calcium crystals in the extracellular matrix and progressive calcification of neointima and lamina media of the vascular wall [82,83]. 

In our lab, we examined the effect of CKD on the vascular circadian clock in two translational models: a model where early uremic vasculopathy, with no calcification, was induced in partially nephrectomized rats that were kept on a high-phosphorus diet for 8 weeks and in a second model where vascular calcification was induced by long-term uremia for 14 weeks together with the administration of a high-phosphorus diet and calcitriol [47,48].

**Figure 2:**
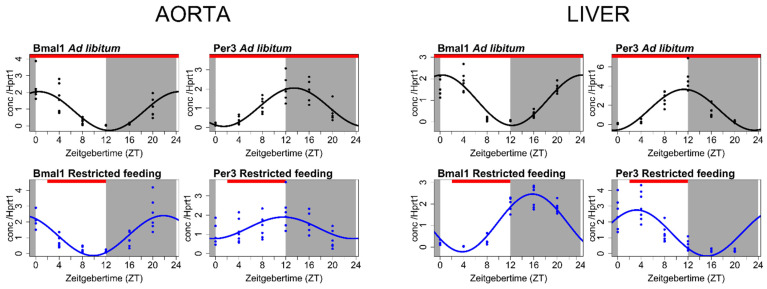
Differential effect of a feeding cue on the circadian clock in the aorta and liver. Many peripheral tissues are known to be entrained by the time of feeding, including the liver, a classical organ primarily responding to feeding cues rather than light input. The input of light and feeding were dissociated by restricting feeding to the habitual inactive period of the nocturnal rats (light period). The effect of restricted feeding on the circadian clock in the rat aorta and in the rat liver are shown. Expression of the core circadian clock genes *Bmal1* and *Per3* in the rat aorta (left) and liver (right) subjected to ad libitum feeding (top row, black, *n* = 38) and after 4 weeks of feeding restricted to ZT2-ZT12 (bottom row, blue, *n* = 39) was examined. As expected, the phase of the circadian clock in the liver was markedly shifted by restricted feeding. In contrast, the phase of the circadian clock in the aorta was not affected by the feeding time. Circadian rhythmicity was assessed by fitting data to a Cosinor regression model (solid lines). Gray areas indicate the dark period, and white areas indicate the light period. Zeitgeber time (ZT; “time-giver”) is the time since light onset. The red bar indicates the feeding time. Gene expression is normalized to the housekeeping gene *Hprt1*.

Primarily, we examined whether an internal molecular clock was operating with circadian rhythmicity in the rat aorta. The aortas were harvested at 4-hour intervals from normal rats and rats at an early stage of CKD. We found in the normal aorta a strong expression of core molecular clock genes: *Bmal1*, *Clock*, *Per1–3*, *Cry1 and 2* and *Rev-Erbα*, all having significant circadian rhythmicity [48] (Figure 2). 

As the timing of food intake may alter the circadian molecular clock in the peripheral tissues, we dissociated lighting and feeding inputs by restricting the food only to be available at the daytime, which is the habitual inactive time period for the nocturnal rat. Then, we examined again the expression profiles of the core circadian clock genes in the aorta after 4 weeks of restricted feeding. The liver was examined as a control organ. The liver is a classic example of an organ entrained primarily by food cues rather than light input to the retina [5,84]. Feeding restricted to the inactive phase markedly shifted the phase of the internal circadian clock in the liver (Figure 2). In contrast, the circadian clock in the aorta performed very robustly and was unaffected by the changing feeding pattern (Figure 2). 

**Figure 3:**
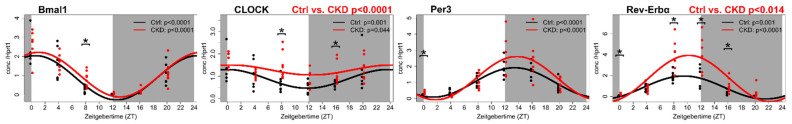
Disturbed expression of circadian clock genes in the uremic aorta after 8 weeks of CKD. Expression profiles of the core circadian clock genes in the aorta after 8 weeks of uremia (red lines) (*n* = 44) are compared to the normal controls (black lines) (*n* = 39). Dots represent each animal. Data are fitted by Cosinor regression, and the resulting *p*-values are shown within the figures. Gray areas indicate the dark period, and white areas indicate the light period. Zeitgeber time (ZT; “time-giver”) is the time since light onset. A significant difference in the Mesor (rhythm-adjusted mean) is indicated on top of the single figures. * Indicates a significant difference (*p* < 0.05) between the groups at specific time points. Data were previously presented in reference [48] and reprinted with permission from Kidney Int.

In early uremic vasculopathy, the significant upregulation of *Rev-Erbα*, *Clock*, *Cry2* and *CK1ε* was found, while *Per1* was downregulated [48] (Figure 3). 

As such, we demonstrated the existence of an internal molecular circadian clock in the rat aorta and found that it is disturbed in uremia. The disturbance of the vascular circadian clock might be involved in the early development of uremic vasculopathy. 

## 5. The Circadian Clock and the Clock-Controlled Genes in the Calcified Aorta of a CKD Rat Model 

In a translational model on long-term CKD and vascular calcification, we examined by RNA-seq analysis the transcriptional profiles of severely calcified aortas in uremic rats [47]. The vascular phenotype of the model included both lamina media calcification and atherosclerotic manifestations. The aortas were harvested between ZT3 and ZT6 (ZT, “*zeitgeber time*” denotes the time since the lights turned on) from CKD rats and compared to those obtained synchronously from the control rats.

Several genes related to the circadian clock were expressed in the normal aorta and were significantly deregulated in the calcified aorta (Table 1). The expression of *Per* genes and of *Dbp*, a *Per1* expression enhancer [85], were significantly downregulated in the calcified aorta, whereas the expression of the transcriptional activator *Clock* was significantly increased in the calcified uremic aorta (Table 1). Among the highly expressed genes in the aorta were *Nr1d1* and *Nr1d2,* which code for REV-ERBα and REV-ERBβ (Table 1). These nuclear receptors cooperate in the regulation of the core clock function, modulating the expression of BMAL1 and further mediating the interplay between the circadian rhythm, metabolism and inflammation [86,87]. The expression of both *Nr1d1* and *Nr1d2* were significantly downregulated in the uremic calcified aorta (Table 1). The significance of REV-ERB for vascular health has been demonstrated in experiments on atherosclerosis-prone LDL receptor-deficient mice, where the administration of a synthetic REV-ERB agonist led to a reduced size of atherosclerotic plaque [88]. REV-ERBα has been shown to modulate the inflammatory function of macrophages through repression of the chemokine C-C motif ligand 2 (*Ccl2*) gene directly through a REV-ERBα-binding motif in the *Ccl2* promotor region, as well as CCL2-activated signals [89]. CCL2 is mechanistically linked to the pathogenesis of many inflammatory diseases, cardiovascular disease and atherosclerosis [90]. Importantly, in a variety of CKD models, the inhibition of the renin–angiotensin system reduced *Ccl2*-mediated inflammation and a pharmacological inhibition of *Ccl2* reduced renal damage [91,92,93,94,95]. Therefore, the strong deregulation of *Nr1d1,* which is found in the uremic calcified aorta, might not only be related to the internal vascular circadian clock but, also, to the inflammatory cellular infiltrates and might, as such, reflect an interesting link between the circadian clock and an inflammatory pathway of importance for the development of vascular calcification in CKD. In this respect, it should be stressed that the expression of *Ccl2*, which is known directly to be controlled by the clock system [96], was significantly upregulated in the calcified uremic aorta [48] (Table 1). 

Proinflammatory cytokines lead to the production of the vascular cell adhesion molecule 1 (VCAM-1) and the intracellular adhesion molecule 1 (ICAM-1). VCAM-1 and ICAM-1 are biomarkers of the endothelial injury and vascular inflammatory processes in CKD [97,98]. The expression of the genes *Vcam1* and *Icam1* was significantly upregulated in the uremic calcified aorta (Table 1). VCAM-1 and ICAM-1 belong to the immunoglobulin (Ig) superfamily of adhesion molecules and are expressed primarily in endothelial cells but, also, in fibroblasts, monocytes and leucocytes [99]. They have an effect on cell adhesion and inflammatory reactions, resulting in the rounding and transmigration of leucocytes through the endothelial layer [100]. Leucocytes are recruited to tissues in a series of consecutive steps from being captured and passing along the endothelial wall, adhesion, intraluminal crawling and transmigration through the endothelial barrier to infiltrate the tissue [100]. In normal homeostasis, leucocytes infiltrate the different tissues in a time-of-day-dependent manner, and both the internal clock in endothelial cells and leucocytes are required for rhythmic homing. The lack of such an internal clock in either cell type is sufficient for disturbing the rhythmic homing [96,101,102]. In mice, endothelial cell-specific BMAL1 deletion resulted in abolishment of the time-of-day fluctuation in the expression of VCAM-1 and ICAM-1 and abrogated the rhythmic homing of leucocytes to peripheral tissues [101]. Whether the circadian rhythm in the endothelial expression of *Vcam1* and *Icam1* is directly mediated via binding of the circadian clock transcription factors to the promoters of these genes is currently unknown. 

Our data suggests a fundamental impact of CKD on the expression of genes related to the rhythmic tissue tropism of inflammatory cells. In addition to diurnal variations in the prevalence of myocardial infarction, the outcome also exhibits time-of-day dependency [22]. A circadian variation in the size of an infarct has been described both in a clinical situation and experimentally [22,103,104,105]. A significantly larger area of the infarct, development of fibrosis and unfavorable remodeling were observed after the onset of ischemia at the sleep-to-wake transition period [106].

The circadian oscillations of neutrophil recruitment to the heart determined the size of the infarct, healing and cardiac function after induction of the infarct [104]. In a healthy heart, a rhythmic recruitment of leucocytes was reported, together with an enhanced adhesion and extravasation of neutrophils, which were paralleled by an increased expression of *Vcam1* and *Icam1* [104]. Whether the deregulated expression of *Vcam1* and *Icam1* found in the calcified uremic aorta reflects the vasculopathy in the vessels of the heart remains to be established. 

Additional mechanisms can potentially link the intrinsic vascular clock to the temporal pattern of cardiovascular disease. The balance between clotting and thrombolysis is known to be affected in a circadian manner [107]. Potentially, the deregulation of the circadian-controlled genes that are responsible for platelet activation, fibrinolytic activity and coagulation contribute to the phenotype of uremic-calcifying vasculopathy. We found that genes coding for the von Willebrand factor (*Vwf*) and thrombomodulin (*Thbd*) were deregulated in the calcified uremic aorta (Table 1). Thus, the expression of *Vwf* was significantly increased in the calcified aortic tissue in CKD. A mechanistic link between the circadian clock and regulation of the von Willebrand factor was previously indicated as a direct regulation of the *Vwf* promoter activity in endothelial cells by the transcriptional regulators CLOCK/BMAL1 [108]. Similarly, the CLOCK/BMAL1 heterodimer was shown to bind to E-box upstream of the *Thbd* promoter and transactivate promoter activity [65]. Thrombomodulin is expressed on vascular endothelial cells and plays an important role in the anticoagulant pathway as a cofactor in the thrombin-induced activation of protein C and also modulates the alternative complement pathway [65]. In the uremic calcified aorta, the expression of *Thbd* was significantly downregulated. 

Several regulators of the cell cycle are under control of the circadian clock [48]. Vascular proliferation contributes to the pathology the uremic aorta. Activated VSMC in the lamina media and intima mediates the proliferation, inflammation and matrix alterations in CKD [44]. In uremic calcified aorta, deregulation of the cell cycle regulators Cyclin D1 and *Wee1*, which are under influence of the circadian clock, has been demonstrated in our lab [47,109] (Table 1).

## 6. Conclusions

**Figure 4:**
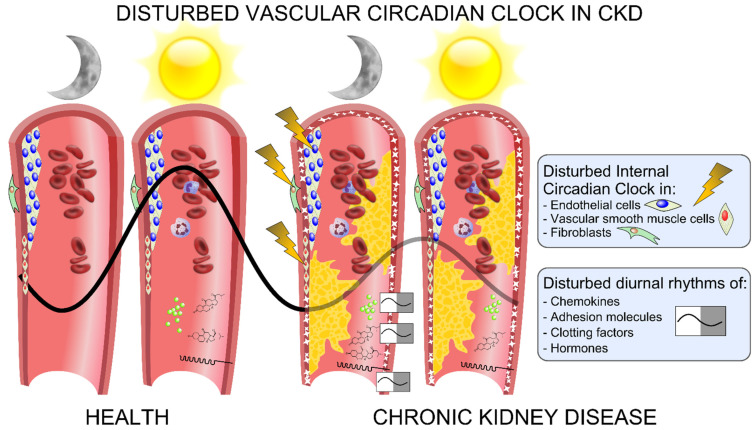
Graphic overview of the disturbances of the vascular circadian clock in chronic kidney disease (CKD). The circadian clock operates in the cells that comprise the vasculature, such as endothelial cells, VSMCs and fibroblasts. In CKD, the vascular circadian clock is disturbed and associated with a disturbance in the diurnal rhythm of chemokines and adhesion molecules such as VCAM-1 and ICAM-1, as well as of clotting factors, blood pressure, hormones and white blood cells, which may contribute to uremic vasculopathy by means of calcification, atherosclerosis, blood clotting, increased migration of immune cells into the vasculature and increased oxidative injury.

The molecular circadian clock is an endogenous self-sustaining pacemaker that operates with a periodicity of 24 h and orchestrates rhythms in the metabolism, hormonal secretion, cell cycle, inflammatory processes and cardiovascular functions. The different properties of the vascular functions are impacted by a circadian clock, which operates in the individual cells of the vascular wall. Disruption of the circadian rhythm may cause serious health problems and may contribute to the development of cardiovascular diseases. Both the master pacemaker of circadian rhythmicity in the hypothalamus and peripheral circadian clocks are disturbed in CKD. In translational models of CKD, the vascular circadian clock is disturbed early in the development, demonstrated in the not-yet-calcified uremic aorta and clearly demonstrated in long-term CKD with vascular calcifying disease. Clock-controlled genes related to the vascular integrity, endothelial function, inflammation and thrombogenesis are severely deregulated in calcified aortas in CKD (Figure 4). There is an urgent need to characterize the impact of this pathology on the malfunction of the vascular system in CKD. Thus, what is the specific input that determines the phase of the molecular circadian clock in the vasculature and how is the clock in the individual cells deregulated in CKD? Translational studies are warranted in order to examine whether targeting the circadian clock might ameliorate uremic vasculopathy and potentially reduce cardiovascular mortality in CKD. 

## Figures and Tables

**Table 1:** Circadian clock and clock-controlled genes in the uremic calcified aorta. Vascular calcification was induced by long-term uremia and a treatment with calcitriol. The aorta gene expressions were analyzed by RNA-seq.

Gene	Name	Control Aorta (rpkm)	Uremic Calcified Aorta (rpkm)	*p*-Value
Bmal1/Arntl	Aryl hydrocarbon receptor nuclear translocator-like 1	7	8	0.52
Clock	Circadian locomotor output cycles kaput	2	5	**<0.001**
Per1	Period circadian clock 1	87	51	**<0.001**
Per2	Period circadian clock 2	51	32	**0.003**
Per3	Period circadian clock 3	16	7	**<0.001**
Cry1	Cryptochrome 1	3	3	1
Cry2	Cryptochrome 2	10	11	0.91
Rev-Erbα/ Nr1d1	Nuclear Receptor Subfamily 1, Group D, Member 1	145	42	**<0.05**
Rev-Erbβ/ Nr1d2	Nuclear Receptor Subfamily 1, Group D, Member 2	10	7	**<0.05**
Dbp	D site of albumin promoter (albumin D-box) binding protein	206	28	**<0.001**
Nfil3	Nuclear factor, interleukin 3 regulated	73	32	**<0.001**
Wee1	WEE1 G2 checkpoint kinase	26	10	**<0.001**
Ccdn1	Cyclin D1	22	86	**<0.001**
Icam1	Intercellular adhesion molecule 1	12	28	**<0.001**
Vcam1	Vascular cell adhesion molecule 1	80	293	**<0.001**
Ccl2	Chemokine ligand 2	2	26	**<0.001**
Thbd	Thrombomodulin	40	23	**<0.001**
Vwf	von Willebrand factor	7	16	**<0.001**

Rpkm: Reads per kilo base per million mapped reads. *n* = 6. RNA-seq data are available in Gene Expression Omnibus under tacking nr.: GSE146638 [47,48]. Data were previously presented in reference [48] and reprinted with permission from Kidney Int.

## Data Availability

Results from RNA sequencing of the calcified rat aorta in CKD compared to control rats have been submitted to The Gene Expression Omnibus, where all data can be found at https://www.ncbi.nlm.nih.gov/geo/query/acc.cgi?acc=GSE146638 (GSE146638) (NCBI tracking system #20700880) Accessed date: 10 March 2020 [47].
